# Linguistic-cultural validation of the oral health assessment tool (OHAT) for the Italian context

**DOI:** 10.1186/s12912-020-0399-y

**Published:** 2020-01-31

**Authors:** Stefano Finotto, Giorgia Bertolini, Riccarda Camellini, Rita Fantelli, Debora Formisano, Maria Grazia Macchioni, Daniela Mecugni

**Affiliations:** 1 Nursing, seat of Reggio Emilia, University of Modena and Reggio Emilia, Campus Universitario “San Lazzaro”, Via Amendola 2 -Padiglione De Sanctis, 42122 Reggio Emilia, Italy; 2 Azienda Unità Sanitaria Locale -IRCCS di Reggio Emilia, Research Nurse Rheumatology, Viale Risorgimento 80, 42123 Reggio Emilia, Italy; 3 Nursing, seat of Reggio Emilia, Department of Surgery, Medicine, Dentistry and Morphological Sciences, University of Modena and Reggio Emilia, Campus Universitario “San Lazzaro” - Padiglione De Sanctis, Via Amendola 2 -, 42122 Reggio Emilia, Italy

**Keywords:** Oral health, Assessment, Elderly adults, Cognitive deficits, Validation

## Abstract

**Background:**

The increase in the ageing population and the consequent establishment of a network of adequate structures to respond effectively to the welfare needs of institutionalized elderly people have stimulated the discussion by healthcare professionals on the subject of oral hygiene.

Literature data show that the same attention has not been paid to oral health care compared to other health needs. Many studies have demonstrated that oral health has a significant impact on the quality of life, especially for older people. Poor oral health also has a considerable role on the physical condition of the elderly because it affects their ability to eat, feed themselves, forcing them to have unbalanced diets. The consequence of this condition is dehydration, malnutrition and impairment of communication skills. The essential nursing activity for oral care is the assessment of the state of oral health, an activity that should be conducted by means of valid tools. To date there are no tools for assessing the health of the oral cavity validated for the Italian linguistic-cultural context. The aim of this study is to conduct a linguistic-cultural validation for the Italian context, of the original Australian version of the *Oral Health Assessment Tool* (OHAT) scale.

**Methods:**

Study design: Linguistic-cultural validation and adaptation of a tool for the assessment of oral health. The Beaton and Sousa & Rojjanasrirat (2011) models were used to conduct the linguistic-cultural validation and adaptation process. This validation involved 368 inmates/patients aged over 65 years with cognitive deficit.

**Results:**

The face validity was confirmed by a score for each item related to clarity equal to or greater than 80%. The content validity was confirmed by an content validity index for items (I-CVI) score equal to or greater than 0.8 for each item and an content validity index for scales (S-CVI) of 0.93 for the entire tool. For the reliability of the internal consistency the Cronbach alpha was calculated, which was found to be 0.82. The test-retest was calculated by means of the *Pearson* coefficient correlation which turned out to be 0.5.

**Conclusions:**

The Italian version of the OHAT is a tool that can help to consider oral health at the same level as other health needs aimed at increasing the quality of nursing care provided. This tool can be used by nurses to assess the health of the oral cavity in elderly subjects also with cognitive deficit.

## Background

The increase in the ageing population and the consequent establishment of a network of adequate structures to respond effectively to its healthcare have stimulated the discussion by healthcare professionals on the subject of oral hygiene since, as reported in the literature, there is evidence that oral healthcare is not delivered at the same level as other health needs [1]. Many studies have shown that oral health has a significant impact on the quality of life, especially for older adults [2–4]. The results of several clinical and epidemiological studies have shown that there is a significant relationship between oral health and specific pathological conditions such as infections of the myocardium, meninges, mediastinum and joint prostheses [5]. Elderly adults who have physical and / or cognitive disorders and who have poor oral hygiene, frequently have a bacterial colonization in the dental plaque biofilm and are exposed to a high risk of aspiration pneumonia. Prevention is based on careful removal of the biofilm from daily dental plaque. However, this practice is often poor or non-existent although it is considered an essential nursing activity [6]. Poor oral health has a significant impact on the physical condition of elderly adults because it affects their ability to eat and feed themselves, obliges them to have unbalanced diets, facilitates weight loss, dehydration and malnutrition, affects communication skills as it impairs the articulation of sound, hinders social relationships as it facilitates the development of pathological behavioural attitudes [7–10]. This is why nurses must not lose sight of the fundamental activities that guarantee an effective response to the needs of this population cohort. In a recent literature review, the concept of *missed nursing care* is presented [11], i.e., the tendency of nurses to prioritize complex activities, thus neglecting the basic care activities, such as assisted personal hygiene and, in particular, oral hygiene. A study by Wardh et al. [12] shows how oral care is considered the most unpleasant task for nurses, probably due to poor oral health training and the necessary support for the implementation of a comprehensive oral care strategy. Sumi et al. [13] also affirm that the topic of oral health is not sufficiently addressed in nursing educational curricula, a statement confirmed by several studies in which nurses have identified for this purpose the need for specific training on oral hygiene [14, 15]. Thorne et al. [16] argue that the effective development of a dental care and oral care service should be based on a regular assessment of all persons accepted in residential care facilities. Since the assessment of oral health is based on the personal ability to report symptoms related to dental suffering, many subjects may have lost this ability, especially if suffering from cognitive deficits, and consequently they are not able to *self-report* data. For these people assessment must be conducted by a qualified dental practitioner or an appropriately trained nurse [17]. In order to facilitate the assessment of oral health, many assessment tools have been developed and validated for non-dental or non-dental health professionals, but they have been designed for a population of patients admitted to intensive care units or in palliative care units, and are therefore unsuitable for use by elderly adults with cognitive impairments in residential care settings [17]. The validated tools for the assessment of oral hygiene in adults with cognitive deficits residing in social-healthcare facilities are: in the United States, the Brief Oral Health Status Examination (BOHSE) [18], in England, The Holistic and Reliable Oral Assessment Tool (THROAT) [19] and, in Australia, the Oral Health Assessment Tool (OHAT) [17].

The BOHSE scale was developed to detect the presence of oral cavity changes in elderly patients in *nursing homes*, with or without cognitive decline and neurological deficit [20]. This scale can be used by nurses who have undergone training conducted by oral health specialists; it consists of 12 categories that include palpation of the lymph nodes of the neck, observation of the lips, inspection of the oral cavity for assessment of the hard palate, the tongue and the gums, salivation, presence of natural teeth, presence of artificial teeth, chewing position and oral cleanliness. These methods require the use of an artificial light source, tongue depressors and gauze for tactile evaluation of any anomalies [18]. The BOHSE scale is a tool meant for screening activities and does not replace the need for a periodic examination performed by a professional dentist. Before using this tool, staff should receive field training from a dentist or dental hygienist.

The THROAT scale involves checking 9 dimensions which include assessment of the lips, presence of dental plaque, changes in the gingiva and oral mucosa, observation of the palate, tongue, sublingual mucosa, smell, consistency of the saliva. So far only a single study has been conducted on the implementation of the scale in a trial that involved a sample of 50 elderly patients; in this study there was no reference to any neurological or cognitive alterations of the elderly patients involved in the trial [19].

The OHAT scale is a re-adaptation of the BOHSE scale. The purpose of this change was to simplify the tool proposed by Kayser-Jones to allow a leaner assessment while maintaining the same criteria of effectiveness. The study of this validation path involved 455 subjects, the results obtained from the evaluation conducted by the nurses compared with those obtained from the evaluation of oral hygiene specialists showed no significant differences. A sufficiently intense artificial or natural light source and the use of disposable gloves were required to perform the assessments; it did not involve the introduction of instruments into the oral cavity or tactile inspection. Compared to BOHSE, assessment of the laterocervical lymph nodes was eliminated.

The OHAT implementation study involves its use on institutionalized elderly people suffering from cognitive and non-cognitive disorders, with varying degrees of dependence [17].

Oral care is particularly important for the elderly population, which, for the most part, has a great health fragility linked to comorbid conditions. The nurse is the reference professional figure taking care of users admitted to long-term care facilities or living in residential facilities for the elderly, and is the person mainly responsible for meeting the healthcare needs expressed by this population cohort. The nursing activity essential for care of the oral cavity is the assessment of the state of oral health. Considering that the literature reports the poor predisposition for the evaluation and care of oral hygiene by nurses, it is essential to plan strategies to implement and ensure this nursing activity.

Seeing that the assessment phase is central in the development of an effective oral care strategy, validated tools are available to the nurses to support them in this activity. It is therefore necessary to provide nurses with a scale for evaluation of oral hygiene also validated in Italian.

## Methods

### Aim

The purpose of this study is the linguistic-cultural validation for the Italian context of the original Australian version in English of the *Oral Health Assessment Tool* (OHAT).

### Study design

Linguistic-cultural validation and adaptation of a tool to assess the health of the oral cavity.

### Method

The Beaton models [21] and Sousa & Rojjanasrirat [22] were employed to conduct the linguistic-cultural validation and adaptation process. The face validity, content validity and reliability of the internal consistency was studied. The criterion validity was not sought, since there are no specific instruments (*gold standard*) in the Italian context. The construct validity was studied by means of factor analysis.

The validation method involves the six phases described below.

#### Phase I

The tool has been translated by two subjects, who possess the characteristics described below, in order to grasp the nuances of language and cultural differences in the most precise way. One of the two translators is bilingual, i.e. is fluent in both the original language - English - of the tool (O) and the target language - Italian - (T); in addition, he/she has profound knowledge of both cultures, as defined by Sousa & Rojjanasrirat [22]. The other translator is a native speaker of the language in which the tool is written - English -, and is also bilingual, as defined by Beaton [21]. One of the translators does not have knowledge of the contents and terminology of the tool, but is familiar with colloquial and commonly used phrases in the target language – Italian, − while the other possesses knowledge about the contents of the tool, as recommended by Sousa & Rojjanasrirat [22]. They have done the work individually and produced a written report, in which they have commented on uncertainties and phrases that are difficult to translate and the cognitive processes that have guided the choices. This phase generated two versions of the tool in the target language (T1 / T2).

#### Phase II

The two translations of the tool were integrated into a single version, in the presence of both translators, a supervisor and members of the study team. The group collaborated in order to compare the discrepancies related to the meanings of words and phrases, between the original version and the two translations, to produce a single version of the tool in the target language (T12). The synthesis process was described in a written report, to document the issues addressed and how they were solved.

#### Phase III

The tool was translated from Italian into the original language, i.e. English, as a process of checking the validity of the tool. The translation, based on the guidelines of Beaton [21] and Sousa & Rojjanasrirat [22], was done individually by two subjects, a native English speaker and obscure to the original version of the tool. One of the translators has no knowledge of the contents and terminology of the tool, but is familiar with colloquial and commonly used sentences in the original language, while the other has knowledge about the contents of the tool. This phase produced two versions of the tool in the original language (O1 / O2).

#### Phase IV

The five versions of the tool (O1-O2-T12-T1-T2) were evaluated by a multidisciplinary committee of experts, in order to achieve trans-cultural equivalence and thereby content validity. This committee was made up of experts in the validation method, nurses and all the translators, involved in the process up to this point. The members of the multidisciplinary committee of experts reached a consensus on all versions of the tool and developed the pre-final version for the field test. The material available to the committee includes the original tool and each translation (O1-O2-T12-T1-T2), together with the corresponding written reports. The committee developed the pre-final version of the tool, verifying the semantic (same meaning of the words), idiomatic (replacement of colloquial expressions and idioms, difficult to translate, with equivalent expressions), experiential (replacing the expressions, which refer to activities not feasible in the culture in which the test will be administered, with equivalent expressions of activity for that culture) and conceptual (evaluation and review of the meaning of concepts that may differ between different cultures) equivalence.

#### Phase V

The pre-final version of the tool was administered to a sample of subjects belonging to the target population, i.e. forty nurses of residential care facilities, and to a group of ten nurse experts in long-term care, to look for the face and content validity and stability of the reliability. To obtain the face validity, the tool was administered asking the nurses to evaluate the indications and elements of the questionnaire using a dichotomous scale (“clear” and “not clear”) and to provide suggestions on how they could rephrase the elements of the tool to make them easier to understand. Subsequently, the percentages of the “clear” and “not clear” answers given for each item were checked: if the item was judged as “not clear” by more than 20% of the nurses, it was re-evaluated; at the end, each item was judged by at least 80% of the sample as “clear”. The tool was also evaluated by a group of ten experts, who know the contents of the tool, the characteristics of the target population and are native speakers of the target language. The experts had to estimate the clarity of the elements of the tool: if the item was judged as “not clear” by more than 20% of the experts, it was re-evaluated; at the end, each item was judged by at least 80% of the experts as “clear”. In the absence of such an agreement it was envisaged that the elements deemed not clear should be reviewed and revaluated. Subsequently, following the models of Beaton [21] and of Sousa & Rojjanasrirat [22], the group of experts evaluated each element of the tool for content validity, using the following Likert scale: insignificant = 1, slightly significant = 2, quite significant = 3, very significant = 4. For reliability of the internal consistency the *Cronbach alpha* was calculated. To find the reliability stability, the tool was administered a second time (after 15 days) to the group of experts, asking them to evaluate each element of the tool using the following Likert scale: insignificant = 1, slightly significant = 2, quite significant = 3, very significant = 4. Reliability stability was calculated using the *Pearson correlation coefficient* (Pearson r*).*

#### Phase VI

This step is used to establish the full psychometric proprieties of the instrument. The sample nurses selected for this phase assessed the health of the oral cavity using OHAT. In this last step the construct validity of the tool was calculated. The tool The exploratory and confirmatory factor analysis was performed.

**Fig. 1:**
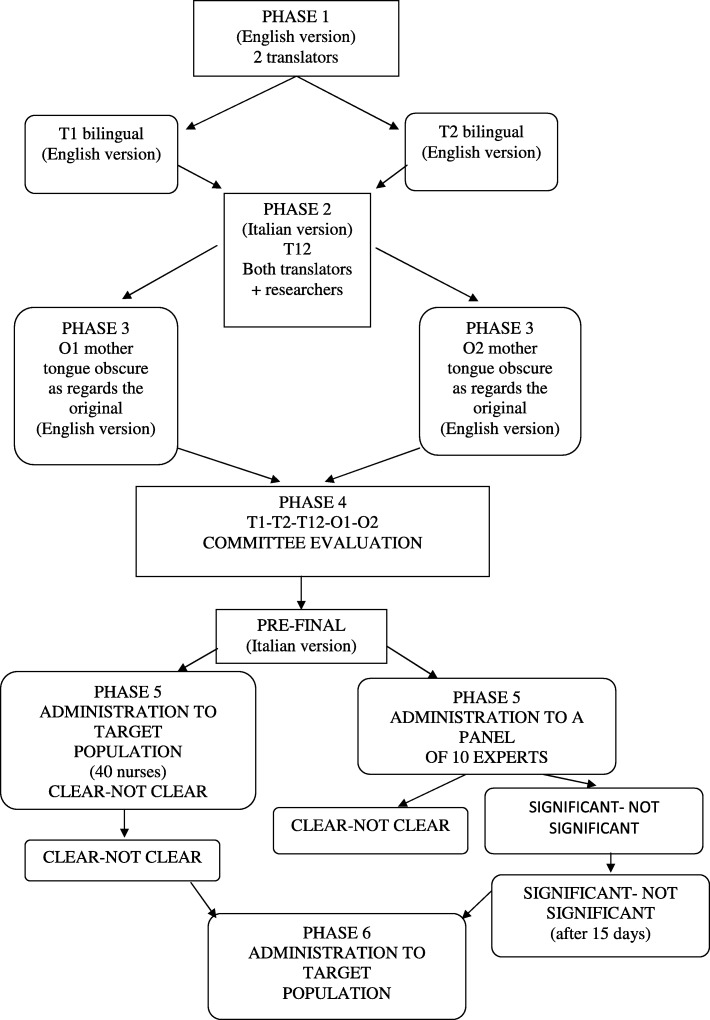
flow-chart of the validation process phases

The six phases of the validation process are shown in Fig. 1.

### Sample

For phase V: the sample of nurses belonging to the target population is of the probabilistic type: forty nurses of the residential care facilities belonging to nursing home (*N* = 20) “ASP Reggio Emilia - Città delle Persone” and of the private clinic (N = 20), “Villa Verde” of Reggio Emilia, affiliated to the National Health Service (Servizio Sanitario Nazionale), were randomized and selected out of a total of 258 nurses. The random number generator of the Emilia Romagna region portal was used for randomization (https://wwwservizi.regione.emilia-romagna.it/generatore/). The sample of experts is of the probabilistic type: ten subjects were randomized and selected, using the random number generator of the Emilia Romagna region portal (https://wwwservizi.regione.emilia-romagna.it/generatore/), from among 30 nursing coordinators of long-term care facilities, geriatrics, medicine departments of the hospital “Azienda Unità Sanitaria Locale” of Reggio Emilia, resident care facilities for the elderly of the nursing home “ASP Reggio Emilia - Città delle Persone”, the private clinic “Villa Verde” in Reggio Emilia and tutors of the Degree Course in Nursing at the University of Modena and Reggio Emilia, seat Reggio Emilia.

For phase VI: the sample was selected for convenience. All the nurses of the long-term care facilities, geriatrics, medicine departments of the hospital “Azienda Unità Sanitaria Locale” of Reggio Emilia, resident care facilities for the elderly of the nursing home “ASP Reggio Emilia - Città delle Persone” and of the medicine and long-term healthcare units of the private clinic “Villa Verde” of Reggio Emilia were included, with assessment of the health of the oral cavity by integrating their data collection tools with the OHAT in 368 inmates/patients aged over 65 years with cognitive deficits.

### Data collection tool

The OHAT was developed based on a tool, the *Brief Oral Health Status Examination* (BOHSE), devised by Kayser-Jones [18] and subsequently modified and used by the Australian Department of Health and Aging (Australian Department of Health and Aging, 2004) and validated by Chalmers et al. [23]. The BOHSE has been modified by simplifying the categories and their content and renamed OHAT [23] .The tool aims to ascertain the state of health of the oral cavity in people living in care facilities with moderate or severe dementia. Its use is intended for those who take care of these people but are not dentists or dental hygienists.

The Italian pre-final version of the Oral Health Assessment Tool consists of eight items, each item can be given a score of 0 = healthy oral cavity, 1 = changes in oral cavity, 2 = unhealthy oral cavity; the score of all the items is added to obtaining a total score. If a score of 1 or 2 appears for any item, a visit to a dentist must be arranged. For this validation study (phases I-V), four items dedicated to the collection of personal data (age, gender, department concerned, seniority) have been added to the eight original items of the tool.

### Data analysis

For the face validity, we calculated the frequencies and percentages. For the content validity, the contents validity index for items (I-CVI) and the scale validity index for scales (S-CVI) were calculated. Three methods can be used for the S-CVI calculation; the one chosen in this study is the averaging method in which average (AVE) means the proportion of items that have had a score of 3 or 4 among the various judges (S-CVI / AVE). It was decided to consider an I-CVI of at least 0.8 and an S-CVI of at least 0.8 as acceptable. The internal consistency reliability was assessed by the statistical measurement of *Cronbach’s alpha*. Stability of the reliability was assessed using the test-retest method, calculating the *Pearson index*. The construct validity was calculated by means of factor analysis. Statistical analysis was performed using EXCEL 2007 software from Microsoft and IBM Statistical Program for the Social Sciences (PASW statistics 18).

## Results

In order to find the face validity of the pre-final version of the OHAT, the tool was administered to the nurses and the experts panel the first time, asking them to judge whether the tool items were clear or not clear and to suggest any corrections. The items received a clarity score of 80% or more and, based on the methodological approach of Sousa & Rojjanasrirat [22], it was not necessary to re-evaluate and modify the items (Table 1).

**Table 1:** points in percentage attributed to the items as regards clear-not clear

Items evaluated as clear at the 1st administration					
Item	Experts	Nurses	Item	Experts	Nurses
1	100	100	19	80	100
2	100	100	20	90	100
3	100	100	21	90	100
4	90	100	22	80	100
5	100	100	23	100	100
6	100	100	24	90	100
7	100	100	25	90	100
8	80	100	26	80	100
9	90	100	27	100	100
10	100	100	28	90	100
11	80	100	29	90	100
12	80	100	30	100	100
13	80	90	31	100	100
14	80	100	32	100	100
15	100	100	33	90	100
16	80	100	34	100	100
17	90	100	35	90	100
18	80	100			

The content validity was evaluated by the experts panel by inserting for each item of the scale a Likert scale that required estimation of the items as insignificant = 1, slightly significant = 2, quite significant = 3 and very significant = 4. Each item obtained an I-CVI equal to or greater than 0.8 (Table 2) and an S-CVI of 0.93 for the entire tool. For the reliability of the internal consistency, the Cronbach alpha was calculated which was found to be .816. For the test-retest, the tool was administered a second time after 15 days to the panel of experts. The correlation coefficient expressed through Pearson’s r was 0.5, showing a fair correlation.

**Table 2:** I-CVI for every item of the pre-final version of the scale

I-CVI for every item			
Item	Experts	Item	Experts
1	1.0	19	1.0
2	1.0	20	1.0
3	0.9	21	0.9
4	0.7	22	0.9
5	0.8	23	0.9
6	1.0	24	0.9
7	0.9	25	0.9
8	0.9	26	0.9
9	0.9	27	1.0
10	1.0	28	1.0
11	1.0	29	0.9
12	0.9	30	0.9
13	1.0	31	1.0
14	1.0	32	1.0
15	0.8	33	1.0
16	0.9	34	1.0
17	0.9	35	1.0
18	0.9		

Finally the construct validity was evaluated by factor analysis. The 8 items correlate at least 0.4 to each other, the measure of adequacy of the sample expressed with the Kaiser-Meyer-Olkin was found to be good with a value of 0.867 (*p* < 0.000). The Bartlett’s sphericity test (χ2 = 814.64, df = 36, *p* < 0.000) indicates that, for the available data, it is appropriate to perform the factorial analysis. Lastly, the communality values were all greater than 0.5, confirming that each item shares its variance with the other items (Table 3). The exploratory and confirmatory factor analysis with varimax rotation showed that the OHAT has two loading factors: internal inspection of the mouth and state of the teeth. At factor 1, internal inspection of the mouth saturates the items lips, tongue, gums and tissues, saliva and oral hygiene; at factor 2, state of teeth saturates the items natural teeth, artificial teeth and dental pain (Table 4).

**Table 3:** communality value of items

Communality value		
	Initial	Extraction
Structure	1.000	.947
Lips	1.000	.640
Tongue	1.000	.578
Gums and tissues	1.000	.633
Saliva	1.000	.646
Natural teeth yes/no	1.000	.735
Artificial teeth yes/no	1.000	.630
Oral cleanliness	1.000	.547
Dental pain	1.000	.494

**Table 4:** exploratory and confirmative factor analysis with varimax rotation

Factor Analysis		
Item	Factor 1 “internal inspection of the mouth”	Factor 2 “state of teeth”
Lips	.775	
Tongue	.744	
Gums and tissues	.750	
Saliva	.742	
Oral cleanliness	.660	
Natural teeth		.813
Artificial teeth		.770
Dental pain		.506

## Discussion

The trans-cultural validation of the research tools is characterized by methodological criticalities, in particular, related to the quality of the translation and the comparability of the results among the different cultural and ethnic groups. This is why it is not enough to translate the questionnaire literally, but it needs to be adapted to make its culturally relevant content comprehensible.

The chosen cultural linguistic validation method is among the most complex if compared, for example, with that described by Sperber [24], in which the pre-final version of the questionnaire is not administered to a sample of subjects belonging to the target population, to find the face validity. The proposal by Sperber [24] does not appear as rigorous as the methodological approach chosen in this study in describing the modalities of translation and the characteristics of translators [25]. Different methods are hypothesized for validation of the translation: evaluation by a group of bilingual experts; focus groups with subjects representing the target population; evaluation by a group of experts that does not include translators who are independent of researchers.

The process of translation of the tool requires skills, knowledge and experience. The colloquial phrases and those characterized by emotional suggestions can be particularly difficult to handle. Moreover, the translation may be formally similar to the original language, but some items may be irrelevant for the target culture, and therefore need to be rephrased or deleted. The methodological approach used should avoid the problems described by Sperber [24] and Hilton [26]; in particular, we should avoid the criticality represented by the exclusive use of bilingual translators, who, as seen from the literature, may have adopted values ​​and attitudes of the culture to which the second language belongs, and may give interpretations different from those of a monolingual translator. On the contrary, it is also possible that the bilingual translator, who has to translate a tool in a language different from the original one, is stimulated by the task of acquiring awareness of the ethnic origin, and consequently to offer ethnocentric interpretations.

The validation process that was developed through the translation of the questionnaire from the source language to the target language produced the Italian version of the tool. The psychometric measures confirmed the validity and reliability of the Italian version of the OHAT. The lowest score, although sufficiently saturated with factor 2 in the factor analysis, concerns dental pain; this is the only item on the scale that concerns a symptom and that it must be reported by the subject that, if suffering from cognitive deficits, he is sometimes unable to express it.

The tool is currently ready to be administered to the target population, i.e. to elderly subjects also with cognitive deficits. As reported in the literature, there is evidence that oral health care is not delivered at the same level as other health needs [1], and that oral health has a significant impact on the quality of life especially for older adults [2–4], constant assessment is essential in order to provide quality care.

**Fig. 2:**
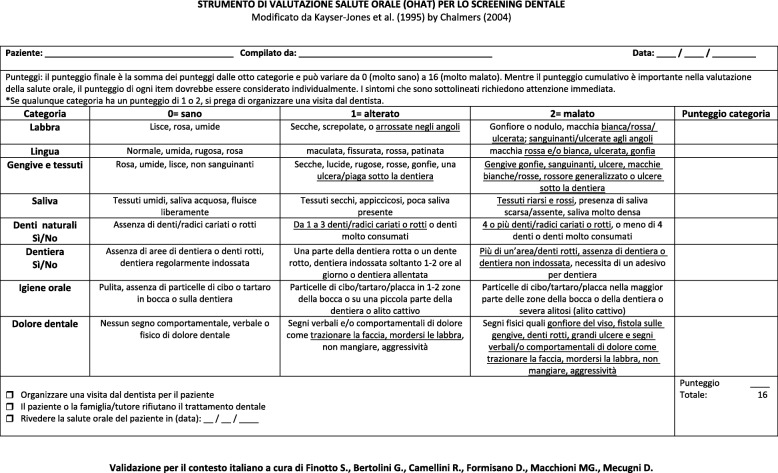
The Italian version of the OHAT

The Italian version of the OHAT (Fig. 2) can help nurses get back some basic assistance activities and contribute to improving the quality of life of elderly subjects also with cognitive deficits by implementing a constant assessment of oral health. The OHAT, in its Italian version, is an easy-to-use tool that requires little time to be administered and that guarantees to identify oral complaints early. For these reasons the instrument assumes a great clinical relevance as it can contribute to decreasing the incidence of complications related to oral health.

## Conclusions

This report describes the linguistic-cultural validation process of a tool for assessment of the state of health of the oral cavity in elderly subjects, also with cognitive disorders, to be used by nursing professionals. The Italian version of the OHAT, the validity and reliability of which has been demonstrated in this study, is a tool that can help to consider oral health as important as other health needs and to increase the quality of nursing care provided.

## Data Availability

The datasets used and/or analysed during the current study are available from the corresponding author on reasonable request.

## Abbreviations

AVE: The proportion of items that have had a score of 3 or 4 among the various judges
BOHSE: Brief Oral Health Status Examination
I-CVI: Content validity index for items
MPS: Mucosal Plaque Index
O1/O2: Versions of the tool in the original language
OHAT: *Oral Health Assessment Tool*
OPI: Ordine delle Professioni Infermieristiche
S-CVI: Content validity index for scales
T1/T2: Versions of the tool in the target language
T12: Single version of the tool in the target language
THROAT: The Holistic and Reliable Oral Assessment Tool
